# Acute Pancreatitis, with Special Reference to Its Treatment, and a Record of Three Cases Presenting Unusual Features

**Published:** 1917-07

**Authors:** Charles A. Morton

**Affiliations:** Professor of Surgery in the University of Bristol; Senior Surgeon to the General Hospital and the Bristol Children's Hospital


### ACUTE PANCREATITIS, WITH SPECIAL REFERENCE TO ITS TREATMENT, AND A RECORD OF THREE CASES PRESENTING UNUSUAL FEATURES.

BY

CHARLES A. MORTON, F.R.C.S.,

*Professor of Surgery in the University of Bristol; Senior Surgeon to the General Hospital and the Bristol Children's Hospital.*

THE diagnosis of acute pancreatitis is but rarely made, for to most surgeons there has not seemed anything really distinctive in the group of symptoms and signs produced by the disease, and its discovery at the laparotomy or in the post-mortem room is generally a surprise. There have been a few cases correctly diagnosed, from time to time, but their proportion to the number of cases in which the diagnosis was not made is very small. Although Moynihan [1] seems justified in saying that although the number of cases that have been diagnosed before operation is extremely small, yet he is

[1] *Abdominal Operations*, 1914, ii. 397.

confident the symptoms are of such a character as to make recognition of the cause a matter of very little difficulty, for he has correctly diagnosed seven out of his last eight cases, yet the fact remains that the diagnosis has rarely been made by other surgeons. Out of a series of six cases reported from University College Hospital[1] by the late Mr. A. E. Barker, in which the patients were under the care of the surgeons of the hospital, one case was diagnosed as perforated gastric or duodenal ulcer, another as intestinal obstruction, while in another no diagnosis could be made. One of the cases was, however, correctly diagnosed by Mr. Raymond Johnson, but in this case the patient's condition was too bad to allow of operation.

The symptoms of acute pancreatitis were described by the American surgeon Fitz several years ago, and his description is quoted by Moynihan in the last edition of his book on *Abdominal Operations.* Within recent years the clinical aspect of the disease has also been very well described by the late Sir F. Eve in his Bradshaw Lecture on "Acute Pancreatitis" in 1915,[2] and by the late Mr. A. E. Barker,[3] and by Mr. Battle.[4] Reference to Fitz's original account or to these writings may with advantage be made for a description of the usual clinical aspect of the disease.

When the abdomen is opened the surgeon usually recognises the true nature of the case by the discovery of white areas of fat necrosis, each about the size of a pin's head, scattered through the subperitoneal fat and the omentum. It is of course well known that these areas of fat necrosis are due to disease of the pancreas, and hence the surgeon's attention is directed to it, and he generally finds it swollen and hemorrhagic, with perhaps some lymph exudation on it,

[1] *Lancet,* 1914, i. 1594. [2] *Ibid.,* 1915, i. 1. [3] *Ibid.,* 1914, i. 1594.
[4] *Ibid.,* 1911, i. 6.

and blood-stained fluid may be found in the lesser sac of the peritoneum, or even in the general peritoneal cavity.

Case 1 is of special interest from the fact that, although when the abdomen was opened the presence of fat necrosis called attention to the condition of the pancreas, yet when the lesser sac of the peritoneum had been opened by division of the gastro-colic omentum, no definitely abnormal condition of the pancreas could be discovered, nor was the pancreas found to be diseased post-mortem until it had been removed from the body, and then some lymph and hemorrhage was seen on its posterior aspect only; and on cutting into it some areas of slight hemorrhage and yellow infiltration were found. The morbid condition was almost confined to the middle of the organ, and although this was the part examined at the operation, none of these morbid changes could have been made out on inspection, or palpation of the presenting aspect of the organ, for they were only discoverable on its posterior aspect.

In Case 2 it is a very interesting and instructive fact that the vomit was fæcal. It is stated in a recent text-book of surgery that the vomit in acute pancreatitis is never fæcal. Certainly fæcal vomiting is most suggestive of obstruction, and was so in this case. The vomit both looked and smelt fæcal.

In Case 3 the great interest of the case was the apparent recovery of the patient after the acute stage. Serious symptoms, however, recurred, and led to a fatal termination. The patient had so far recovered from the acute stage, at the end of which she was admitted (which seemed probably due to the passage of gall-stones or their retention in the common duct), that I discussed with her the desirability of having an operation for removal of gall-stones before another attack came on, and yet at that time the acute pancreatitis must have been passing into the sloughing stage. Though hemorrhagic, gangrenous, and suppurative pancreatitis are by some writers described as different forms of the disease, they are more frequently regarded as only different stages, for the condition of acute hemorrhagic pancreatitis is found to end in suppuration or gangrene in a certain number of cases. If the disease is present in its most severe form at the onset, very likely the patient will not survive to the gangrenous or suppurative stage, and it is of course quite possible that resolution of the acute stage may take place without either suppuration or gangrene.

The condition of temporary improvement after the acute stage of the disease was almost as marked in one of the six cases collected by the late Mr. Barker at University College,[1] as in my own case. After a very acute onset, and the discovery of acute pancreatitis at operation, by the third week there was "much general improvement, and the progress was generally favourable," and then, as in my case, symptoms recurred, and sloughs began to discharge through a drainage tube which had been introduced from the loin to the region behind the pancreas. In the second case recorded in this series there was also rapid improvement up to the eleventh day after draining the lesser peritoneal sac, and then symptoms recurred, and a collection of blood and pus had to be drained from the left side of the abdomen two days later.

In all three of my cases, as in other published cases, there was a history of recurrent attacks of pain in the upper abdomen. What was the nature of these attacks? They were not followed by jaundice. In one of the cases the gall-bladder contained a large stone, and in another many small stones, and in the third no stones were found in it, or in the common duct. It is unlikely that if the attacks in case No. 3, in which no stones were found in the gall-bladder, had been due to the passage of gall-stones, that every stone should have

[1] *Loc. cit.*

been passed before the onset of the fatal illness. Therefore, in this case at any rate, the recurrent attacks were probably not of the nature of gall-stone colic, and it is doubtful if they were in the other two.

**Case 1.**—A woman, E. P., aged 42, was seen in consultation with Dr. Cook at 1.30 p.m., Thursday, November 21st, 1907. Her illness began on the previous Sunday (the 17th), with pain across the upper abdomen and vomiting, and the pain had continued in the same position, but there had been only very occasional vomiting since the day of onset. The bowels had acted. For a fortnight before the onset of the marked abdominal pain on the 17th there had been slight pain in the same region. During the earlier part of the year she had had four attacks, in which Dr. Cook had attended her, and he thought they had all been similar to the present one. There had been no jaundice in any of them.

She was a very fat woman, and there were several inches of fat in the abdominal wall, but apart from this there was no marked distension of the abdomen. The upper half of the abdomen was very tender, and the tenderness was not only epigastric, but was present in each hypochondriac region, and was very marked in the right one. The lower abdomen seemed quite normal, and pelvic examination was negative. A little later in the afternoon after her admission to hospital her temperature was 101°, pulse 110 and of good strength, respiration 22. By that time the pain had increased

With the history of the attacks of pain in the upper abdomen, it seemed possible there might be some cholecystitis associated with gall-stones. I did not suspect the presence of acute pancreatitis. But her condition seemed serious enough to call for immediate operation, which I performed at 4 p.m. I opened the abdomen through the right semilunar line, and explored the gall-bladder. It contained a very large stone. Numerous areas of fat necrosis were seen in the omentum, which directed attention to the pancreas. This was exposed by another incision in the linea alba, with division of the gastro-colic omentum. When I had thus exposed the pancreas, I could not discover anything abnormal about it, nor any hemorrhage in the surrounding tissues. The day following the exploration she was free from abdominal pain, but suffered from frequent vomiting of dark fluid, and became collapsed and died at the end of twenty-four hours from the operation. The temperature fell to normal after the operation, and remained so until it rose to 101.6° again a few hours before death. Rectal and then subcutaneous saline had no very definitely beneficial effect.

At the post-mortem examination fat necrosis was found to be very marked in the omentum, and in the subperitoneal fat in the region of the pancreas. Nothing abnormal could be made out in the condition of the pancreas until it had been removed from the body, and then some lymph exudation and hemorrhage was seen on the posterior surface. On making a section of the organ, an area of slight hemorrhage and purulent infiltration were discovered in the centre. The head and tail of the pancreas were almost free from the morbid process. The organ was not obviously enlarged, and the amount of hemorrhage was very slight, hardly more than blood-staining. Nothing abnormal could be seen on the anterior surface. No gall-stone was present in the common duct, and there was no pancreatic concretion.

**Case 2.**—A man, J. L., aged 58, was sent to me at the General Hospital by Dr. Lewis, of Winscombe, on March 28th, 1914. He had had attacks of pain in the right upper abdomen for four years, but not at frequent intervals. A year at one time, and six months at another, had intervened between the attacks. The last attack had been eight weeks before admission. There had been no jaundice after any of the attacks, and there was, when I examined him, no tinge of bile in the conjunctiva or urine. I could feel nothing of the gall-bladder, but he was tender in the middle line high up in the epigastrum. It was not evident that the attacks were of the nature of gall-stone colic, though this was suspected, and if they were, the attacks hardly seemed to occur often enough to call for operation, and he returned home. But two days after he returned home he was attacked by severe epigastric pain, and this continued. He vomited on the following day, but only once, and Dr. Lewis sent him into hospital again on the evening of the third day, *i.e.* on April 3rd. When I saw him at 11 a.m. on April 4th I found that distinct fæcal vomiting had set in since admission, and his pulse was 120 and feeble. There had been a slight liquid fæcal action and some gas passed as the result of an enema, but from the time of the onset of the pain to his admission enemata had failed to produce any action of the bowels. There was a very slight tinge of bile in the conjunctiva, and also in the urine. There was moderate distension of the abdomen, but this was almost confined to the upper part. It was not rigid, but the upper part was tense and hyper-resonant. There was no swelling anywhere, and rectal examination was negative. There was no rise of temperature. The quite marked fæcal character of the vomit very strongly suggested mechanical obstruction, and I wondered, taking into consideration the previous history, whether it might be obstruction by a gall-stone.

I operated at noon, after emptying the stomach of its fæcal contents. The abdomen was opened through the upper part of the right rectus. There was a large quantity of brown serum in the general peritoneal cavity, especially in the right kidney pouch. Numerous minute areas of fat necrosis were present in the omentum and the sub-peritoneal fat, and the condition was confluent in the region of the head of the pancreas. The pancreas was considerably enlarged and very hard. No hemorrhage was seen. He was pulseless at the end of the operation, but the pulse returned after intravenous saline infusion with pituitary gland extract; he lived only for a few hours

At the post-mortem examination the pancreas was found to be greatly enlarged, and of a purple colour. There was a large area of hemorrhage in the centre, and the head and tail were of a pink colour and swollen. There was also hemorrhage into the surrounding tissue. There were many stones in the gall-bladder, and one very minute fragment of stone in the common duct. The upper part of the small intestine was distended with fluid right up to the duodeno-jejunal junction, but the lower part of the ileum was quite empty. There was no mechanical obstruction and no peritonitis. The fæcal vomiting was no doubt due to the distension of the upper part of the small intestine, but there was nothing to explain the cause of this distension.

**Case 3.**—A woman, J. W., aged 64, was admitted to hospital on Monday, December 11th, 1916, and seen by me that evening. During the six months before admission she had four attacks of epigastric pain passing through to the back, with vomiting, and each lasted some hours. The present illness had begun on Thursday, December 7th. like one of the other attacks, but the pain had persisted for four days, and the vomiting had been very frequent during that period. She had required one hypodermic injection of morphine for the pain on one of the four days. The bowels had not been open during the illness, and an enema after admission had had no result.

She was a very fat woman, and had a great thickness of fat in the abdominal wall, but the abdomen was not distended or rigid, and was only slightly tender in the epigastrium, and to the left of it. She had no abdominal pain. Pulse was 80 and quite gocd, and temperature 100°. When seen by daylight next morning it was doubtful if there was any bile-tinge in the conjunctiva. There was no definite bile reaction in the urine.

The bowels acted freely after hot turpentine enemata. The slight epigastric tenderness disappeared. She was at first fed by nutrients only, but there was no recurrence of any vomiting after admission. Her temperature was 100° the evening of the day after admission, and then quite normal for five days, and she seemed to be making quite a satisfactory recovery from what seemed probably a very prolonged attack of gall-stone colic, but no stones could be found in the motions. But on the 16th the symptoms recurred. Abdominal pain and vomiting again set in, and the abdomen became tender and rigid. Her pulse was at first raised to 112, and later became very rapid and so feeble as to be uncountable, and she was very restless and wandering. The temperature was not at first raised, but at 2 p.m. on the 17th she had a rigor, and it ran up to 105°. There was a slight jaundiced tinge in the conjunctiva, but no bile in the urine. The rigor suggested to my mind the presence of stone in the common duct, or from the serious condition of the patient perhaps a portal infection. She had another rigor and temperature of 105° on the 18th, and after remaining below 100° on the 19th and 20th, she had a third rigor on the 21st , and the temperature again rose to 105°. But her condition very much improved after the 17th. The abdominal pain, tenderness and rigidity passed off, though she still vomited occasionally, and her pulse was greatly improved by subcutaneous saline infusion and fell to 70–80. Her condition seemed sufficiently improved to make an exploration for stone in the common duct desirable, though she was still very feeble, and her tongue very dry. The slight tinge of jaundice in the conjunctiva persisted. The repeated rigors, with a marked improvement in her condition, were even more suggestive of a stone in the common duct, for the improvement seemed to negative portal pyæmia.

I operated on December 24th. I did not find any stones in either the gall-bladder or common duct, but the head of the pancreas felt much enlarged. It was wrapped up in a mass of very fat omentum, and in the omentum were numerous areas of fat necrosis. I regarded the case as one of subacute or chronic pancreatitis, although fat necrosis was much more suggestive of the acute disease, and I drained the gall-bladder, and did not interfere with the pancreas itself. She was in very fair condition at the end of the operation, but she was very drowsy next day. Several times she vomited. An attempt was made by subcutaneous saline infusion to improve her failing pulse. The temperature ran up to 104.6° at 10 p.m. on the 25th, but there was no rigor. Bile was discharged freely from the tube in the gall-bladder. She died on the early morning of the 26th.

At the post-mortem examination, on drawing up the transverse meso-colon, and separating some coils of small intestine from its right side, near its root, a small cavity was opened, which contained necrotic tissue, and into it gas and liquid fæces welled up from the bottom. On further examination this cavity was found to lie over a gangrenous pancreas, with which it communicated. The whole central part of the pancreas was in a condition of greenish black pulp, and a large slough of pancreatic tissue represented the head, and was almost detached, and another replaced the tail. The intestinal perforation was from necrosis of the duodenal wall, where it passed round the head of the pancreas. This necrosis was very extensive, and had caused a collection of fæcal matter to form in the left loin beneath the panceas. No stone was present in the common duct. There was no sign of peritonitis in the general peritoneal cavity, and no free fluid.

We must recognise the fact that sometimes, when nothing more is done than an exploratory laparotomy, a patient suffering from acute pancreatitis may recover. An instance of this has been recently published.[1] There is no reason to think the laparotomy in any way contributed to the patient's recovery, indeed it would rather tend to prevent it, but without any exploratory laparotomy it would hardly be possible to say that acute pancreatitis had been present in cases of recovery. It is, of course, quite possible that some cases of acute abdominal disease that recover without operation may be instances of acute pancreatitis.

I venture to think that all surgeons can do in acute pancreatitis is to provide drainage for exudation into the lesser sac of the peritoneum, or into the retro-peritoneal tissue around the pancreas. Inflammatory exudation into the lesser sac is of course drained through the abdominal incision, but suppuration and sloughing of the pancreas is obviously best drained from behind, though an anterior drainage tube passing through the peritoneal cavity is better than no drainage at all. But posterior drainage is rather a formidable undertaking in a patient so ill as one suffering from acute pancreatitis. As the pancreas extends much more to the left of the spine than the right, the drainage should be on left side, but in order to reach the pancreas there, we have to burrow from the loin through a region full of large vessels. If we study the anatomical relations of the pancreas, we find that the right place to burrow is between the upper part of

[1] *Brit. Med. Journ.*, March 17th, 1917, p. 359.

the left kidney and the spleen, through an incision below the last rib in the loin, and then as we work forwards we have the splenic vessels between our fingers and the pancreas. It would, I think, be very dangerous to burrow with even sinus forceps, which might tear the splenic vein, and the only safe way would be to work gently with the finger-tip. The late Mr. A. E. Barker made such an incision on the right side, but he had one hand in the abdomen to guide him, and he said "it was not as difficult as he expected." This case teaches us that when we establish such posterior drainage, it is well not to shorten the tube for some considerable time, for suppuration and sloughing may be late in their onset.

One difficulty in making a satisfactory posterior drainage opening in these cases, when the surgeon uses one hand on the anterior aspect of the pancreas in the abdominal cavity, as a guide to the other hand burrowing towards the pancreas from a posterior incision, is that the skin of the loin is not prepared for operation, as the disease is probably only discovered as the result of the laparotomy, and it would be a very undesirable proceeding to operate from behind through unprepared skin with the peritoneal cavity open. And another difficulty is that with the opened abdomen, the patient must of course be rolled on the side to get at the loin. I think the best plan to meet both these difficulties is to close temporarily the abdominal incision, and cover it with a sterile towel; then roll the patient over on to the side, and prepare the skin of the loin with some rapid method such as cleaning the skin, and then applying eusol or iodine, after first drying it with spirit; then to make the loin incision and burrow in the direction of the pancreas, and then when we imagine that it is nearly approached turn the patient partly on the back again, open up the abdominal wound, and then insert the fingers of the left hand on to the pancreas, as a guide to the burrowing finger of the other hand in the loin wound. If the patient is too bad to stand this somewhat complicated operation, we must be content with anterior drainage by leaving a large drainage tube reaching from the abdominal wall down to the pancreas, but should establish posterior drainage at a later date, before sloughs begin to separate, unless the symptoms subside completely after the anterior drainage, but we must remember that a period of apparent recovery may mislead us, as it did in Case 3.

Incision into the pancreas is recommended in these cases of acute pancreatitis, either in the form of small incisions or multiple punctures, but it seems to me that though such incisions or punctures may certainly relieve tension, and allow of the escape of inflammatory exudation, yet if they are going to act efficiently in this way the incisions must go deeply into the gland, and that would involve considerable risk from hemorrhage. The late Mr. A. E. Barker, in speaking of incision of the pancreas, said "it seemed risky to incise the swollen area, and allow the inflammatory products to escape into the peritoneal cavity and drain through it," and his case recovered with posterior drainage alone. In the other successful cases of Mr. Barker's series,[1] the pancreas was not incised.

In Cases 1 and 2 drainage, or incision into the gland, would not have saved the patients' lives. In Case 3 drainage might have done good, but at the time I operated the toxæmia was too profound and too quickly fatal to make it at all probable that it would have led to the patient's recovery. When I was able to determine that a lesion of the pancreas was present in this case, from the finding of the fat necrosis and the swollen condition of the head of the pancreas, I had no knowledge of the extensive gangrene which was discovered at the post-mortem examination. I seemed to me most likely that the pancreatitis was subacute, and associated with an undiscoverable stone in the common

[1] *Loc. cit.*

duct, to the presence of which the signs had been due. Perhaps it is as well that I did not further explore the condition of the pancreas by opening through the very fat-laden gastro-colic omentum, for I should by so doing probably have disturbed the adhesion of the coil of small intestine to the under aspect of the transverse meso-colon, and thus have infected, perhaps without discovering it, the general peritoneal cavity.

It is suggested that if in operating on a case of acute pancreatitis gall-stones can be felt in the gall-bladder, they should be removed and the gall-bladder drained, if it does not too seriously prolong the operation. Unless we had evidence that the removal was almost essential to the patient's recovery, it seems to me that we should not prolong the operation by removing gall-stones or draining the gall-bladder. It is not the presence of stones in the gall-bladder, but stones in the common duct which is likely to cause pancreatitis, and even if we discover one there, in the presence of this acute and dangerous disease its removal could hardly be wisely undertaken at that time. In Case 3 I drained the gall-bladder because I did not realise how advanced and serious was the pancreatic disease, and it is only indicated in the less serious and more chronic form of the disease.

